# A Comparative Analysis of Transfected and Integrated Auxin Reporter Systems Reveals Sensitivity Advantages in Protoplast Transient Expression Assays

**DOI:** 10.17912/micropub.biology.001481

**Published:** 2025-02-28

**Authors:** Joseph S. Taylor, Eric A. Villaseñor, James Rashkovsky, Jaime Simson, R. Clay Wright, Bastiaan O. R. Bargmann

**Affiliations:** 1 School of Plant and Environmental Sciences, Virginia Tech, Blacksburg, Virginia, United States; 2 Department of Biological Systems Engineering, Virginia Tech, Blacksburg, Virginia, United States

## Abstract

Reporter-gene activation studies using transient transformation of protoplasts are a powerful tool for the investigation of transcriptional regulation in plants. Here, we perform a comparative analysis of reporter-gene activation sensitivity using an integrated versus a co-transfected reporter-gene construct in Arabidopsis seedling mesophyll protoplasts. The DR5 synthetic auxin-responsive promoter was used to assay the response to auxin treatment and over-expression of activator Auxin Response Factors. We show that sensitivity, as measured by the fold-change in fluorescent-protein reporter-gene expression, is significantly increased by using a co-transfected reporter-gene construct.

**Figure 1. Flow-cytometric analysis of reporter-gene activation:**
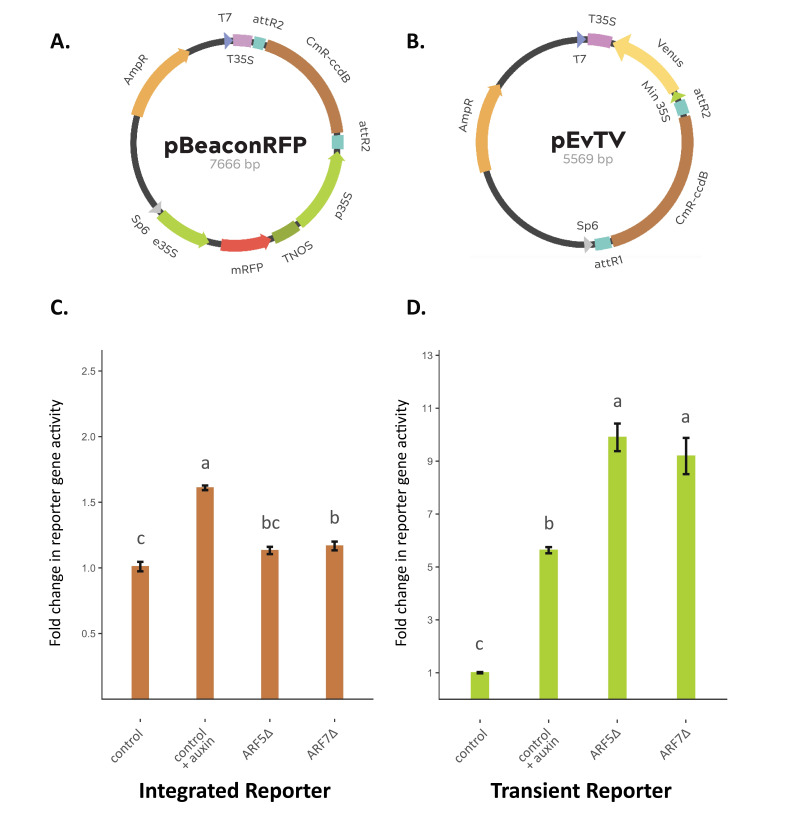
A) A schematic representation of the pBeaconRFP transient effector construct, a plasmid containing a 35S-driven mRFP positive marker and Gateway cassette. B) A schematic representation of the pEvTV transient reporter construct, containing a Gateway cassette connected to a minimal 35S-driven Venus. C-D) pBeaconRFP_GUS control and ARFΔ effector constructs were transfected into Arabidopsis mesophyll protoplasts containing the stably integrated DR5rev::GFP reporter gene (C) or wild-type mesophyll protoplasts co-transfected with the pEvTV_DR5 reporter construct (D). Reporter activity is presented as fold change relative to the untreated control. +auxin samples were treated with 100 nM of IAA overnight. Transfections and treatments were performed in triplicate; statistical significance was assayed by one-way ANOVA followed by a Tukey post hoc test; bars indicate standard error, letters indicate homogeneous subsets (p < 0.05; n=3). &nbsp;

## Description

Reporter-gene activation studies using transient transformation of protoplasts are a powerful tool for the investigation of transcriptional regulation in plants. Some of the prominent advantages of this technique are the speed and throughput with which experiments can be conducted as well as the ability to have a quantitative read-out of reporter-gene activation.

These assays have been used extensively in the study of auxin signaling. One of the earliest examples of its use is the investigation of the auxin-responsive GH3 promoter (Liu et al., 1994) . This study made use of protoplasts derived from carrot cell-suspension cultures that were transformed with two plasmids: one with the auxin-responsive reporter-gene constructs driving the expression of β-glucuronidase (GUS) and one with the constitutive 35S CaMV promoter control construct driving the expression of luciferase. To account for differences between various transfections and promoter constructs, the GUS activity measured for each transfection was normalized by dividing it by the luciferase activity from that same sample. Both were measured by assaying cell lysate using a fluorimeter and luminometer, respectively.

Subsequent studies using this technique to study the auxin signaling pathway included a third plasmid, the effector construct that used a constitutive promoter to drive the expression of transcriptional activators or repressors to test their effect on reporter-gene activation, e.g., (Ulmasov et al., 1997a, 1997b) . Later work transitioned to the use of Arabidopsis mesophyll protoplasts (Tiwari et al., 2001) . Later still, investigations into factors influencing auxin-responsive reporter-gene activation switched to using a reporter gene that was integrated into the genome of stably transformed Arabidopsis transgenic lines, as opposed to a co-transfected reporter-gene construct (Wang et al., 2005; Tiwari et al., 2006) .

Pivoting away from enzymatic reporter-gene assays, we developed the use of flow-cytometric quantification of fluorescent reporter-gene activation in individual protoplasts a few years later (Bargmann and Birnbaum, 2009) . This technique made use of an effector plasmid (pBeaconRFP; Figure 1A ) that simultaneously expresses a positive fluorescent selection marker (red fluorescent protein; RFP) along with the effector-of-interest and measures the output of an integrated reporter gene consisting of an auxin-responsive promoter driving the expression of a green fluorescent protein (DR5rev::GFP (Benková et al., 2003), henceforth, DR5::GFP). This technique allowed for the flow-cytometric quantification of reporter-gene activation only in the successfully transformed (RFP-positive) protoplasts with the transfection of just one plasmid. So far, we have applied this approach primarily to protoplasts derived from Arabidopsis seedling roots (Bargmann and Birnbaum, 2009; Yu et al., 2015; Gonzalez et al., 2021) . Additionally, we have also seen strong activation of a co-transfected DR5::Venus reporter-gene construct (pEvTV_DR5; Figure 1B ) as measured by flow-cytometric quantification in cannabis mesophyll protoplasts (Beard et al., 2021) .

In recent projects, we have shifted to using transient transformation of Arabidopsis mesophyll protoplasts over those from seedling roots for auxin-responsive reporter-gene activation assays. This has been motivated by the ease of obtaining larger quantities of mesophyll protoplasts compared to seedling root protoplasts and the ability to study reporter-gene activation in a more homogeneous cell population. However, we noticed that, in our hands, the activation of the integrated DR5::GFP reporter gene was less sensitive in mesophyll protoplasts compared to root protoplasts. Therefore, we set out to test whether the use of a co-transfected reporter-gene construct allows for a more sensitive readout in Arabidopsis mesophyll protoplasts.

Protoplasts were prepared from the cotyledons and leaves of seven-day-old seedlings, either wild-type (Col-0) or transgenic lines carrying the integrated DR5::GFP reporter gene (also in the Col-0 background). After isolation and purification, the protoplasts were transfected with pBeaconRFP plasmids with various effectors and, in the case of co-transfection experiments in the wild-type seedlings, the pEvTV reporter construct with the DR5 promoter. The pBeaconRFP constructs were made to express either GUS (as an inert control that should not affect the auxin response) or truncated versions (Δ) of two class A Auxin Response Factor (ARF) transcription factors, ARF5 ( AT1G19850 ) or ARF7 ( AT5G20730 ). These truncated ARFs lack the C-terminal PB1 protein interaction domain and therefore fail to interact with the natively expressed Aux/IAA repressors. In essence, ARF5 Δ and ARF7 Δ are gain-of-function, or irrepressible, ARFs that are expected to activate the transcriptional auxin response even in the absence of auxin (Krogan et al., 2012; Wu et al., 2015; Gonzalez et al., 2021) . Some of the pBeaconRFP_GUS transfected control cells were also treated with 100 nM auxin (indole-3-acetic acid; IAA) overnight, as a positive control to elicit the auxin response.

The next day, the fluorescent properties of the transfected protoplasts were measured by flow cytometry. The GFP or Venus intensity as a measure of reporter-gene activation was quantified in the RFP-positive cells and expressed relative to the untreated control. In protoplasts with the integrated reporter gene, auxin treatment led to a 1.61-fold increase in reporter-gene activity, the expression of ARF5 Δ and ARF7 Δ led to a 1.13- and 1.17-fold increase, respectively ( Figure 1C ). In protoplasts co-transfected with the pEvTV_DR5 reporter-gene construct, auxin treatment led to a 5.63-fold increase in reporter-gene activity, while expression of ARF5 Δ and ARF7 Δ led to a 9.9- and 9.19-fold increase, respectively ( Figure 1D ). These results show that the use of the co-transfected reporter-gene construct significantly increased the reporter-gene sensitivity to auxin treatment as well as transiently expressed effectors.

In conclusion, we were able to achieve a more sensitive readout of reporter-gene activation in Arabidopsis mesophyll protoplasts co-transformed with a reporter-gene construct relative to an integrated reporter system. This allowed us to perform experiments using more easily obtained protoplasts (from seedling leaves, compared to seedling roots) that consisted of a more homogeneous cell-type population (mostly mesophyll cells, compared to the heterogeneous population make-up found in root-derived protoplasts). Hence, we were able to circumvent the issue of the auxin response, as measured by an integrated DR5::GFP reporter gene, being less sensitive to auxin treatment in leaf-derived protoplasts. For reference, 50 nM IAA treatment and ARF5 Δ expression resulted in a 2.1- and 1.7-fold change in Arabidopsis seedling root protoplasts with an integrated DR5::GFP reporter gene, respectively (Gonzalez et al., 2021) . We ascribe this increased sensitivity to the availability of many more copies of the reporter gene per cell. This may also explain why we see a higher activation by the ARF5 Δ and ARF7 Δ effectors relative to the auxin treatment in the co-transfected cells, as there is less competition with native ARF proteins for promoter binding. It is possible that some increase in the signal we observed can be ascribed to the fact that Venus is a brighter and more rapidly maturing fluorophore than GFP. However, we would expect this effect to be consistent and proportional across auxin treatments and effector treatments, which is not the case. The nearly two-fold increase above auxin treatments of the ARF5 Δ and ARF7 Δ effectors with the transient pEvTV_DR5 reporter is in stark contrast with the integrated reporter for which the response to these effectors is barely above the GUS control. This strong and specific reporter response will facilitate future high-throughput functional studies of transcriptional effectors and their interactions with promoters.

## Methods

*Plant Growth*

Arabidopsis seeds (wild-type Col-0 and DR5rev::GFP [ABRC stock CS9361 (Benková et al., 2003)]) were surface sterilized by 7 min incubation with 70% [v/v] ethanol, followed by 14 min incubation with 20% [v/v] household bleach, and one wash with sterile water. The seeds were densely plated on 10x10 cm square media plates (2.2 g/L Murashige and Skoog salts with vitamins, 1% [w/v] sucrose, 1% [w/v] agar, pH 5.7). The plates were incubated in a plant growth chamber [Conviron, Canada], oriented horizontally, at 21 °C with 18-hour photoperiod of 100 µmol*m ^-2^ *s ^-1^ PAR.

*Plasmid Preparation*

DNA plasmid purification for pBeaconRFP_GUS (Gonzalez et al., 2021) and pEvTV_DR5 (Lieberman-Lazarovich et al., 2019) was outsourced to GenScript Biotech, Inc. [USA]. Plasmid DNA for all other constructs were purified from Mach1 T1 *E. coli* grown in 50 mL Luria Broth with 50 µg/mL carbenicillin for 16 hours at 37 °C and extracted using the QIAGEN plasmid extraction midi kit [QIAGEN, USA].

Table 1: Plasmids

**Table:** 

Plasmid	Description
pBeaconRFP_GUS	Gateway vector (empty vector available via the VIB Vector Vault, Vector ID: 3_20) that encodes a 35S-driven mRFP protein and GUS to serve as a transformation control. GUS encodes for β-glucuronidase P05804 (UniProt).
pBeaconRFP_ARF5Δ	Gateway vector (empty vector available via the VIB Vector Vault, Vector ID: 3_20) that encodes a 35S-driven mRFP protein and ARF5Δ. ARF5Δ is derived from ARF5 ( AT1G19850 , TAIR10) and lacks the C-terminal Phox and Bem 1 (PB1) interaction domain, i.e., all amino acids beyond T794.
pBeaconRFP_ARF7Δ	Gateway vector (empty vector available via the VIB Vector Vault, Vector ID: 3_20) that encodes a 35S-driven mRFP and a truncated ARF7Δ. ARF7Δ is derived from ARF7 ( AT5G20730 , TAIR10) and lacks the C-terminal PB1 domain, i.e., all amino acids beyond residue T1039.
pEvTV_DR5::Venus	pEvTV gateway vector carrying the DR5 promoter driving Venus. Sourced from Dr. Idan Efroni (Hebrew University of Jerusalem Israel).

&nbsp;

&nbsp;

*Protoplast Isolation, Transformation, and Treatment*

Cotyledons and leaves of seven-day-old seedlings were harvested and placed into a 250 mL flask with 50 mL enzymolysis solution (1.25% [w/v] Cellulase R-10 [Yakult, Japan], 0.3% [w/v] Macerozyme R-10 [Yakult, Japan], 0.4 M mannitol, 20 mM MES, 20 mM KCl, 10 mM CaCl _2_ , 0.1% [w/v] bovine serum albumin; pH was adjusted to 5.7 with Tris-HCl pH 7.5) and shaken at 50 rpm for five hours. After five hours, the shaking was turned up to 100 rpm for an additional 20 minutes. The solution was then filtered through a 40 µm Falcon cell strainer (VWR, Radnor, PA, USA), divided over 15 mL conical tubes, and centrifuged for 5 min at 120 g with no braking. The cell pellets were resuspended in 3 mL enzymolysis buffer (no enzymes added) and then floated on top of a sucrose cushion (6 mL of 0.6 M sucrose, 2 mM MES, pH 5.7) in a 15 mL conical tube and centrifuged again for 7 mins to pellet out debris. The cell layer was extracted and washed in enzymolysis buffer, counted with a hemacytometer, and centrifuged again for 5 min in a 15 mL conical tube. Cells were washed once with transfection buffer (0.4 M mannitol, 15 mM MgCl _2_ hexahydrate, 4 mM MES; pH was adjusted to 5.7 with KOH), centrifuged again, and resuspended in transfection buffer with a final density of 6 × 10 ^5^ protoplasts*mL ^−1^ . Conical 15 mL tubes were prepared for each transfection, each containing 250 µL of protoplasts (approximately 1.5 x 10 ^5^ cells) in transfection buffer and the appropriate plasmid DNA (Table 2). Protoplasts with the integrated DR5::GFP reporter were transfected with 37.5 µg of the pBeaconRFP_GUS vector or 25 µg pBeaconRFP_ARF5∆ or pBeaconRFP_ARF7∆ plus 12.5 µg of the pBeaconRFP_GUS vector (Gonzalez et al., 2021) . In the case of co-transfections with the pEvTV_DR5 vector, wild-type protoplasts were transfected with 25 µg of the pBeaconRFP_GUS, pBeaconRFP_ARF5∆, or pBeaconRFP_ARF7∆ vector plus 12.5 µg of the pEvTV_DR5 vector. This ensures that the total amount of DNA used remained equal across all conditions (Table 2). In accordance with Gonzalez et al. (2021), 250 µL PEG solution (40% [w/v] PEG 1500, 0.2 M mannitol, 0.1 M CaCl _2_ ) was added, and the suspension was mixed by flicking the tube repeatedly. Suspensions were incubated for 1 min, after which the protoplasts were washed with 15 mL enzymolysis buffer, centrifuged, and resuspended in 1 mL enzymolysis buffer. Protoplast suspensions were divided in 24-well plates (1 mL per well), treated with 100 nM indole-3-acetic acid (or mock treated), and incubated for 17 hours in the dark at 21 °C. Three independent transformations were performed for each construct combination and treatments were performed in triplicate.

Table 2: Transfections and Treatments

**Table:** 

Name	Integrated DR5::GFP Reporter	Wild-type Col-0
Control	37.5 µg pBeacon_RFP_GUS	25 µg pBeacon_RFP_GUS 12.5 µg pEvTV_DR5:Venus
Control + Auxin	37.5 µg pBeacon_RFP_GUS 100 nM IAA (treatment)	25 µg pBeacon_RFP_GUS 12.5 µg pEvTV_DR5:Venus 100 nM IAA (treatment)
ARF5Δ	25 µg pBeacon_RFP_ARF5Δ 12.5 µg pBeacon_RFP_GUS	25 µg pBeacon_RFP_ARF5Δ 12.5 µg pEvTV_DR5:Venus
ARF7Δ	25 µg pBeacon_RFP_ARF7Δ 12.5 µg pBeacon_RFP_GUS	25 µg pBeacon_RFP_ARF7Δ 12.5 µg pEvTV_DR5:Venus

*Flow Cytometry*

The GFP or Venus intensity in RFP-positive cells was quantified using a SH800S cell sorter (Sony Biotechnology Inc., USA) with forward-scatter and side-scatter, 488 nm excitation and 525/50 nm emission for GFP/Venus (green fluorescence), 561 nm excitation and 617/30 nm emission for RFP (red fluorescence). The population of live cells in a forward scatter-area vs. side scatter-area plot was identified by back-gating RFP-positive events from a green vs. red fluorescence plot on a forward scatter-area vs. side scatter-area plot and drawing a “live-cells” gate that encompassed 90% of the RFP-positive events in the forward scatter-area vs. side scatter-area plot. This “live-cells” gate was then used to distinguish live cells from debris in the forward scatter-area vs. side scatter-area plot and used to generate a green vs. red fluorescence plot enriched for live cells. Using this live-cells green vs. red fluorescence plot, RFP-positive cells were identified and GFP/Venus fluorescence intensity (arbitrary units) was quantified solely in this population. Statistical significance was assessed by one-way ANOVA and subsequent Tukey post hoc testing to identify homogeneous subsets ( *p* < 0.05, n = 3 biological replicates).
